# The transition of pemphigus vulgaris into pemphigus foliaceus: A case report

**DOI:** 10.1016/j.jdcr.2026.06.060

**Published:** 2026-07-15

**Authors:** Anna Stasiewicz, Agnieszka Grocholska, Alicja Frączek, Natalia Zdanowska, Agnieszka Owczarczyk-Saczonek

**Affiliations:** a School of Medicine, Collegium Medicum, University of Warmia and Mazury, Olsztyn, Poland; b Clinic and Department of Dermatology, Sexually Transmitted Diseases and Clinical Immunology, School of Medicine, University of Warmia and Mazury, Olsztyn, Poland

**Keywords:** autoimmunity, desmoglein compensation, pemphigus foliaceus, pemphigus vulgaris, subtype transition

## Introduction

Pemphigus comprises a group of autoimmune blistering diseases mediated by IgG autoantibodies directed against the desmoglein 1 (Dsg1) and desmoglein 3 (Dsg3), which are key keratinocyte adhesion molecules located in desmosomes. Pemphigus vulgaris (PV) and pemphigus foliaceus (PF) are the 2 main clinical variants. PV is further divided into mucosal-dominant and mucocutaneous subtypes, reflecting distinct anti-Dsg1 and/or anti-Dsg3 autoantibody profiles with painful blisters and erosions, primarily in the oropharyngeal mucosa and/or skin. In contrast, PF is characterized by autoantibodies directed against Dsg1 and the absence of mucosal involvement.^1^ A shift between these subtypes, first reported in 1991 by Iwatsuki et al,^2^ suggests an unusual evolution of the patient’s immune response. To date, 50 cases of PV-to-PF transition have been reported.^3^^,^^4^ Here, we present the case of a patient diagnosed with PV, which subsequently developed into PF after 13 years.

## Case report

A 73-year-old woman presented to the Dermatology Clinic with a 2-week history of eroded and crusted plaques in seborrheic areas: face, chest, and back. The medical history included surgically and chemotherapeutically treated ovarian and endometrial carcinoma (1998), type 2 diabetes mellitus, hypertension, hypercholesterolemia, and PV. Notably, a prior diagnosis of PV had been established in 2012. This diagnosis was originally confirmed via direct immunofluorescence, demonstrating intercellular IgG deposition in a “fishnet pattern,” indirect immunofluorescence with a pemphigus antibody titer of 1:320, and positive enzyme-linked immunosorbent assay (ELISA) results for anti-Dsg1 and anti-Dsg3 autoantibodies. Previous therapeutic interventions included methylprednisolone, prednisolone, and cyclophosphamide, with remission. Treatment was ended in July 2021, with the last documented disease activity in 2016. On admission, the patient was receiving rosuvastatin (5 mg), spironolactone (25 mg), losartan (50 mg), empagliflozin (10 mg), sitagliptin (100 mg), nicergoline (10 mg), amlodipine (5 mg), and insulin aspart.

**Fig 1:**
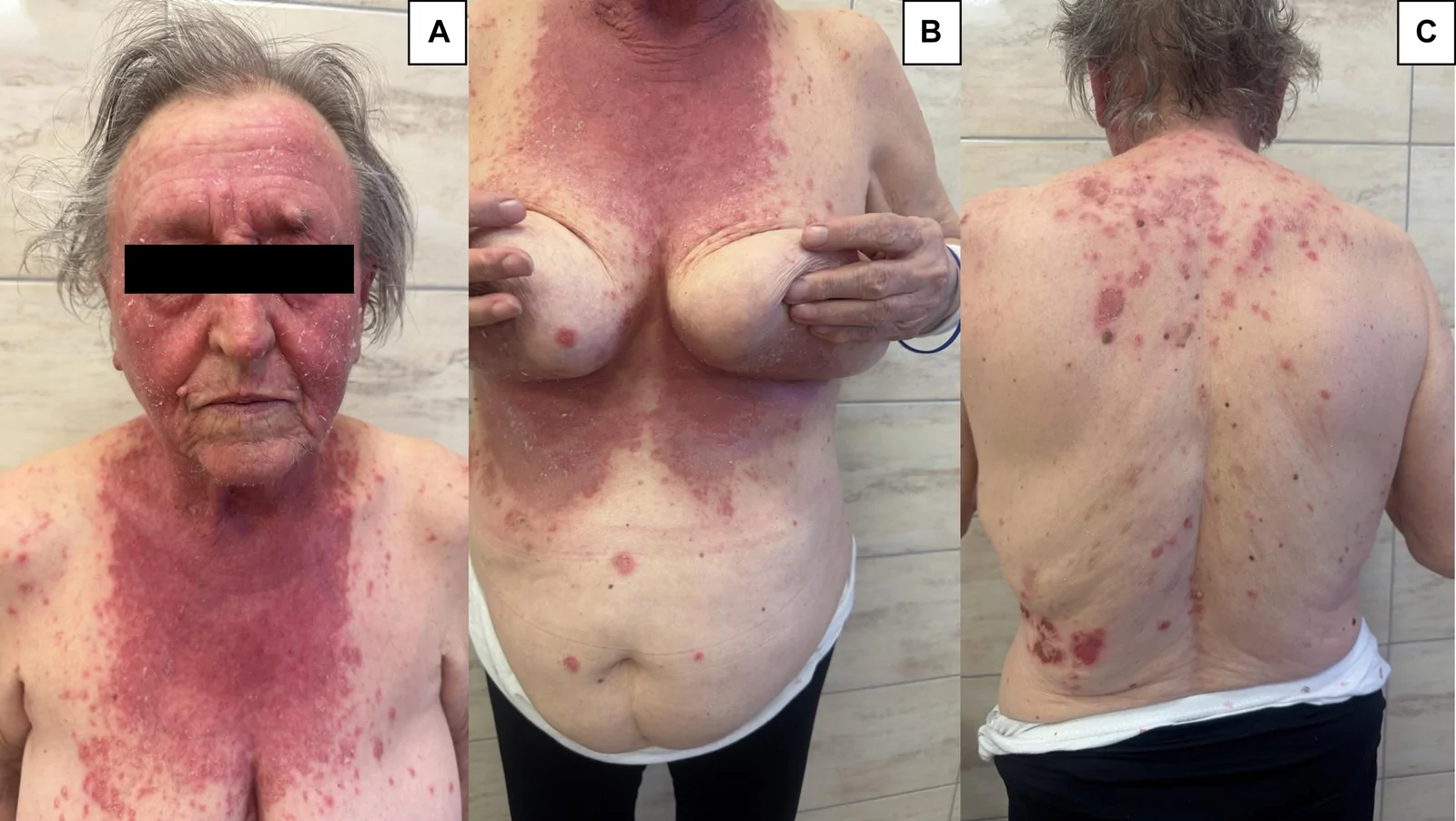
Pemphigus foliaceus. Skin lesions presented by the patient during hospitalization. **A,** Confluent erythematous-infiltrative-exfoliative lesions on the face and chest. **B,** Intertriginous lesions with lichenification under the breasts. **C,** Erosion on the skin of the back.

Physical examination at admission revealed confluent erythematous, infiltrative, and exfoliative plaques involving the face and chest (Fig 1, *A*). Beneath the breasts and in the axillae, similar lesions were observed, accompanied by lichenification (Fig 1, *B*). On the trunk, particularly across the upper and lower portions of the back, scattered erosions partially covered with scales were present (Fig 1, *C*). No mucosal involvement was found.

During hospitalization in August 2025, laboratory investigations revealed elevated levels of C-reactive protein (38.2 mg/L), elevated levels of carbohydrate antigen 19-9 (30.8 U/mL), and proteinuria (69.4 mg/dl). Direct immunofluorescence demonstrated that intercellular IgG deposits within the epidermal intercellular spaces. ELISA showed elevated anti-Dsg1 antibody levels with negative anti-Dsg3. Antinuclear antibody titer was 1:320, with no prior data available. Immunoblot was negative. Based on the clinical presentation and serological profile, a diagnosis of PF was established.

**Fig 2:**
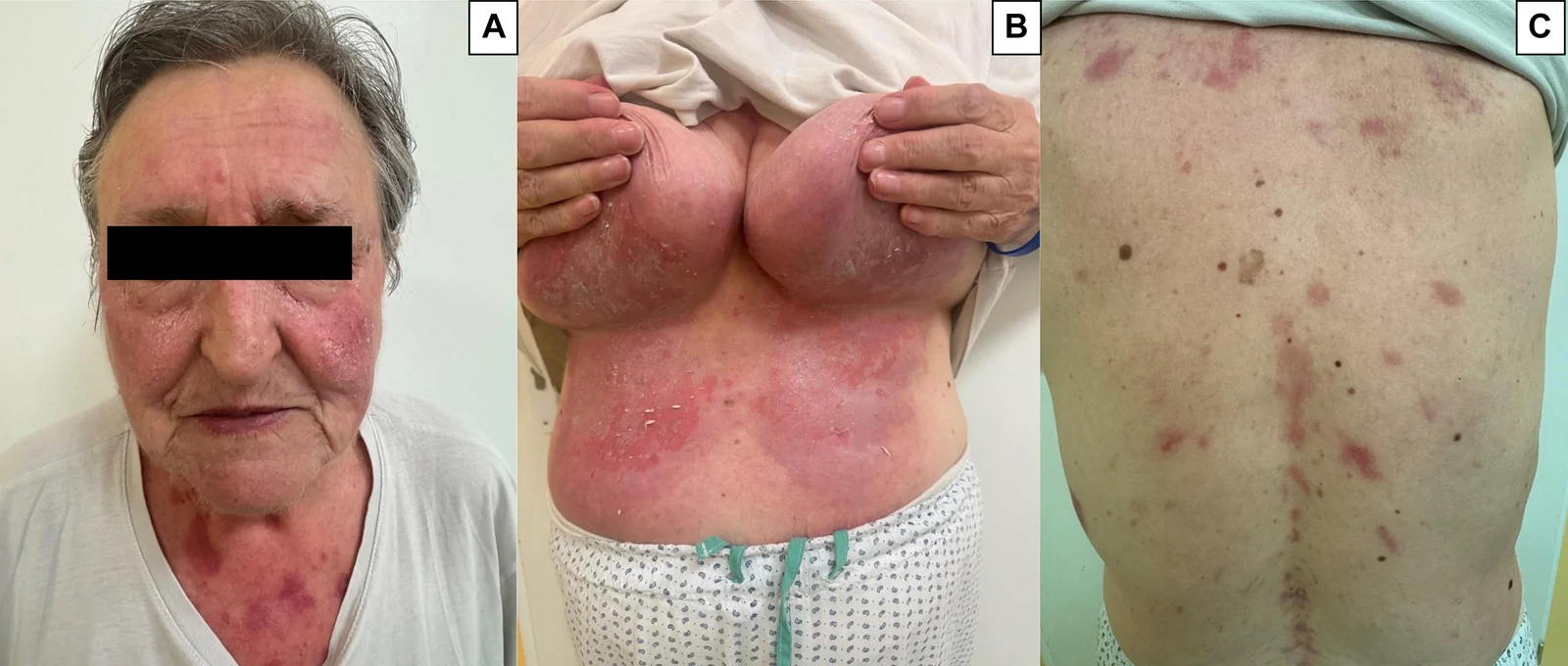
Pemphigus foliaceus. Skin lesions following 10 days of treatment. **A,** Reduction of erythema and exfoliation on the face and chest. **B,** Regression of intertriginous lesions beneath the breasts. **C,** Healed erosions on the back with residual postinflammatory hyperpigmentation.

The patient was treated with intravenous methylprednisolone pulses (250 mg × 3 days), followed by oral prednisone (30 mg daily) and azathioprine (100 mg daily), along with topical clobetasol and clotrimazole. After 10 days of hospitalization, there was a significant clinical improvement (Fig 2, *A-C*).

The scheduled follow-up was missed, with the patient returning 4 months later in December 2025. Laboratory examinations and abdominal ultrasound revealed no changes compared with the previous hospitalization; however, antinuclear antibody titer decreased to 1:160. A subsequent course of methylprednisolone pulse therapy was administered. Although the following hospitalization was scheduled for January 2026, the patient has remained noncompliant.

## Discussion

The clinical progression observed in our patient, characterized by initial PV (anti-Dsg1 and anti-Dsg3) followed 13 years later by a PF phenotype (anti-Dsg1 only), represents a documented but rare pemphigus subtype conversion.

The mechanism underlying this shift remains incompletely understood. Didona and Di Zenzo^5^ proposed that epitope spreading may contribute to the PF-to-PV transition. According to that model, severe PF-related tissue damage related to anti-Dsg1 autoantibodies may expose hidden Dsg3 epitopes, eliciting anti-Dsg3 autoantibody production and subsequent mucosal involvement characteristic of PV.^5^ However, the shift observed in our case would hypothetically require a different immunological process, namely, the loss or preferential down-modulation of the anti-Dsg3 response with persistence of anti-Dsg1.^3^^,^^4^

España et al^6^ analyzed antibody profiles during PV-to-PF transition and demonstrated that pathogenic antibodies directed against the amino-terminal extracellular domain of Dsg1 persist throughout the conversion, whereas antibodies against the EC1 and EC2 domains of Dsg3 undergo marked reduction or complete loss, correlating with lack of mucosal lesions. This pattern indicates that subtype shift involves selective suppression of the anti-Dsg3 response.

The factors responsible for the disappearance of anti-Dsg3 antibodies remain unknown, as does whether it is permanent or reversible, therapy-induced phenomenon. To date, 3 cases of drug-related pemphigus subtype conversion have been reported: 1 attributed to rilzabrutynib and 2 to rituximab.^7^, ^8^, ^9^ Our patient had received long-term prednisone therapy as management of PV, which could have preferentially depleted high-affinity anti-Dsg3 memory B cells and T cells while anti-Dsg1 clones recovered or persisted.^6^ Nonetheless, subtype transitions have also been documented in patients who did not receive immunosuppression, indicating that such conversions can occur independently of pharmacologic intervention.^5^

Drug-induced PF should also be considered in the differential diagnosis in such presentations. PF has been associated with angiotensin-converting enzyme inhibitors, angiotensin II receptor blockers, calcium channel blockers, thiazide-like diuretics, and β-blocker-thiazide combinations.^10^ In our patient, the chronic medication regimen included losartan (angiotensin II receptor blocker) and amlodipine (calcium channel blocker), both implicated in PF triggering. However, the well-documented prior PV diagnosis and the long-term use of these agents suggest that a drug-induced etiology is less likely than a genuine subtype conversion.

Furthermore, an appropriate diagnostic approach is essential to avoid misclassification. Pemphigus diseases are typically identified through evaluating clinical presentation with histologic and immunohistochemical analysis.^1^ In reported cases of PV-to-PF conversion, authors applied different techniques, including histopathology, direct immunofluorescence, indirect immunofluorescence, and desmoglein ELISA.^3^^,^^4^ Definitive subtype classification requires serological confirmation through antigen-specific assays, most preferably anti-Dsg1 and anti-Dsg3 ELISA, which also enables quantitative monitoring of disease activity and treatment response.^1^^,^^11^

In the management of pemphigus, systemic corticosteroids remain the primary therapeutic approach. Current guidelines recommend oral prednisone as the treatment of choice in limited disease, while in moderate-to-severe cases, prednisone is combined with azathioprine, mycophenolate, mofetil, or rituximab, an anti-CD20 antibody. Other immunosuppressive agents, biologic drugs, and intravenous immunoglobulins are reserved for refractory cases and rare subtypes.^1^ In our patient, intravenous methylprednisolone pulse therapy was applied on the basis of the presence of comorbidities and the necessity of achieving rapid disease control.

This case confirms that subtype transition from PV to PF represents a clinically plausible phenomenon, although the underlying factors remain largely unknown. Further prospective studies and long-term patient observation are required to define the mechanisms and frequency of this rare conversion. ELISA testing is vital for identifying and monitoring such shifts, enabling precise subtype characterization. Despite distinct serological profiles, the therapeutic approach does not differ substantially between pemphigus subtypes, as corticosteroids combined with adjuvant immunosuppressive therapy remain the treatment of choice in all clinical entities.

## Conflicts of interest

None disclosed.
